# In Situ Copolymerized Polyacrylamide Cellulose Supported Fe_3_O_4_ Magnetic Nanocomposites for Adsorptive Removal of Pb(II): Artificial Neural Network Modeling and Experimental Studies

**DOI:** 10.3390/nano9121687

**Published:** 2019-11-25

**Authors:** Imran Hasan, Rais Ahmad Khan, Walaa Alharbi, Khadijah H. Alharbi, Ali Alsalme

**Affiliations:** 1 The Environmental Research Laboratory, Department of Chemistry, Chandigarh University, Mohali 140301, India; 2 Department of Chemistry, College of Science, King Saud University, Riyadh 11451, Saudi Arabia; aalsalme@KSU.EDU.SA; 3 Department of Chemistry, Faculty of Science, King Khalid University, P.O. Box 9004 Abha, Saudi Arabia; 4 Department of Chemistry, Science and Arts College, Rabigh Campus, King Abdulaziz University, Jeddah 21911, Saudi Arabia

**Keywords:** polymer grafting, artificial neural network, optimization, Langmuir, BET surface area

## Abstract

The inimical effects associated with heavy metals are serious concerns, particularly with respect to global health-related issues, because of their non-ecological characteristics and high toxicity. Current research in this area is focused on the synthesis of poly(acrylamide) grafted Cell@Fe_3_O_4_ nanocomposites via oxidative free radical copolymerization of the acrylamide monomer and its application for the removal of Pb(II). The hybrid material was analyzed using different analytical techniques, including thermogravimetric analysis (TGA), Fourier transform-infrared spectroscopy (FTIR), X-ray diffraction (XRD), scanning electron microscopy (SEM), transmission electron microscopy (TEM), and Brunauer–Emmett–Teller (BET) analysis. The efficacious impact of variable parameters, including contact time, pH, material dose, initial Pb(II) concentration, and the temperature, was investigated and optimized using both batch and artificial neural networks (ANN). Surface digestion of metal ions is exceedingly pH-dependent, and higher adsorption efficiencies and adsorption capacities of Pb(II) were acquired at a pH value of 5. The acquired equilibrium data were analyzed using different isotherm models, including Langmuir, Freundlich, Temkin, and Redlich–Peterson models. In this investigation, the best performance was obtained using the Langmuir model. The maximum adsorption capacity of the material investigated via monolayer formation was determined to be 314.47 mg g^−1^ at 323 K, 239.74 mg g^−1^ at 313 K, and 100.79 mg g^−1^ at 303 K.

## 1. Introduction

Two examples of critical global issues include ecological assurance and vitality supply [1,2]. The release of inorganic contaminants such as metals into groundwater from a variety of sources, including metal plating, refineries, and agrarian activities, has resulted in adverse health outcomes in humans and has negatively impacted the biological community [3,4,5]. The deleterious impact of these metals is considered to be a genuine global biohazard due to their non-ecological properties and toxicity [6]. Among the various heavy metals, Pb(II) is the most basic metal, is widespread, and is exceptionally poisonous, causing maladies such as cancer and adverse effects in individuals, even at low levels [7,8]. As such, there has been considerable research on the development of eco-friendly, efficacious, and viable techniques for the sequestration of heavy metals. Examples of ongoing research related to this problem include precipitation [9], particle trade [10], electro-dialysis [11], layer filtration [12], and adsorption [13]. Among these strategies, adsorption has been classified as the most promising strategy for wastewater treatment because of the high osmosis rate and simplicity of the approach, without causing adverse side effects [14].

Recently, biopolymer-based joined copolymer nanocomposite materials have attracted considerable worldwide attention from specialists and researchers because of their broad applications, including for the treatment of wastewater. Naturally occurring polymers, including alginate, starch, cellulose, dextrin, chitosan, and guar gum, have also attracted significant interest among scientists, particularly with respect to heavy metal sequestration. This interest is primarily due to their uncommon biochemical properties, accessibility, and the presence of different functional groups on the backbone of the polymer chain [15,16]. Cellulose, a straight polymeric attachment of the β-1,4-connected D-glucopyranose monomer, is an abundant polysaccharide that is chiefly utilized as a fundamental component of paper, materials, and films [17]. Cellulose is an extremely promising low-cost crude material for heavy metal sequestration because of its desirable properties, including biodegradability, sustainability, biocompatibility, and non-toxicity [18].

Among the different metals and metal oxide nanoparticles, magnetic oxide nanoparticles (MNPs), such as Fe_3_O_4_, are unique materials with numerous potential applications in physical, concoction, and organic sciences [19]. From the onset, MNP-based strong stage extrication has been considered to be a viable procedure for limiting both natural and inorganic analytes in complex media [20]. Fe_3_O_4_ nanoparticles are the most widely utilized materials in magnetic solid phase extraction (MSPE) because of their functional density, magnetic character, low toxicity, and the simplicity of their blend. On the other hand, polymers are also promising materials compared to magnetic nanoparticles MNPs and are also potentially suitable for the treatment of various toxins in water [21]. Poly(acrylamide) (PAM), a water–solvent hydrogel polymer made of acetyl amine groups on a macromolecular spine, can interact with metal particles via substance or physical connections [22,23,24,25,26].

Artificial neural networks (ANN) have emerged as important statistical tools for the optimization and prediction of complicated functional variables in the sequestration process [27]. Various fields of science and engineering have adopted these techniques. ANN models can be applied as alternative mathematical approaches to solve a wide variety of problems, such as process control, system recognition, and point prediction [28]. These statistical tools can be applied to a variety of problems in research and development to optimize chemical reactions with respect to different process variables.

In this article, PAM chains were joined onto cell@Fe_3_O_4_ nanoparticles to improve the scattering property of cell@Fe_3_O_4_ (Poly(acrylamide)-g-Cell@Fe_3_O_4_, PAC@Fe_3_O_4_) in water and to enhance the adsorption limit by increasing the surface thickness. The new material was characterized using different techniques, including Fourier transform-infrared (FTIR) spectroscopy, X-Ray diffraction, thermogravimetric analysis (TGA), Brunauer–Emmett–Teller (BET) analysis, scanning electron microscopy (SEM), and transmission electron microscopy (TEM). The material was also investigated in terms of the removal of Pb(II) from water. The combined effectiveness of variable adsorption parameters were investigated, including fomentation time, arrangement pH, adsorbent portion, starting metal particle fixation, and temperature, and were advanced using consistent group strategies. Finally, the optimization process was further examined via the application of an artificial neural network.

## 2. Experimental Section

### 2.1. Chemicals

Microcrystalline cellulose and acrylamide (Sigma-Aldrich, Banagalore, India), FeCl_3_·6H_2_O and FeCl_2_·4H_2_O (Merck, Bangalore, India), liquor alkali (Fischer Scientific, Mumbai, India), and nitrate salts of separate metal particles were utilized as obtained, without further sanitization. Azobisisobutyl nitrile (AIBN) was acquired from Merck India. Stock solutions of 1000 mg L^−1^ were prepared by dissolving an appropriate amount of nitrate salt of the metal particle of interest in deionized (DI) water.

#### Amalgamation of Cell@Fe_3_O_4_ Nanoparticles

The nanoparticles were blended using a straightforward one-stage substance co-precipitation technique [29]. In summary, 5.0 g of FeCl_2_·4H_2_O and 10.0 g of FeCl_3_·6H_2_O were added to 150 mL colloidal cellulose (5.5 g) in DI water, while mixing continuously at a speed of 950 rpm for 3 h. Subsequently, 30 mL of 25% ammonia solution (NH_4_OH) was gradually added after the solution was warmed to 80 °C. Mixing was continued for 3.0 h at 80 °C under a consistent mixing and nitrogen environment. The resulting product was washed with DI water five to six times to remove any unreacted species, before drying at 60 °C.

#### Fabrication of Poly(acrylamide)-g-Cell@Fe_3_O_4_ (PAC@Fe_3_O_4_) Nanocomposite

**Figure 1:**
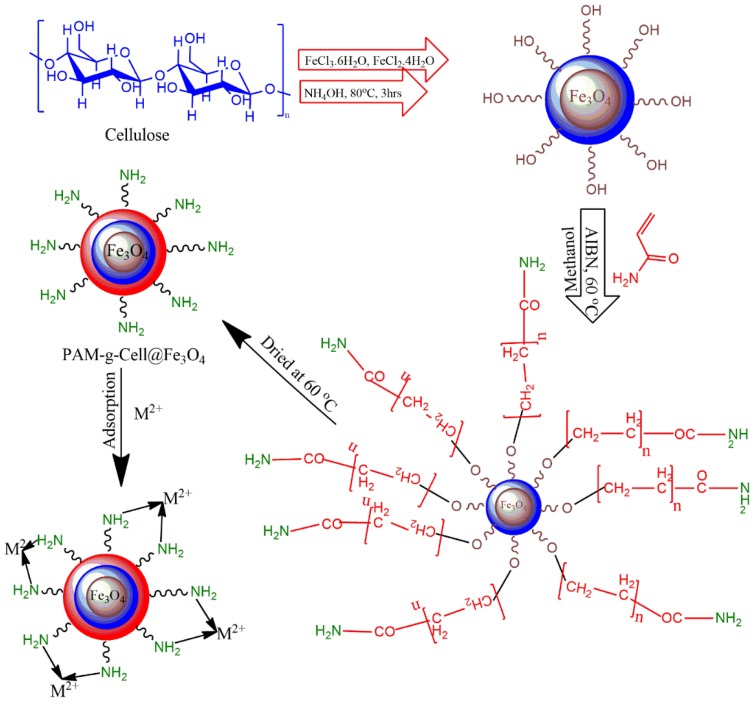
Proposed scheme for synthesis and functioning of poly(acrylamide)-g-Cell@Fe_3_O_4_ (PAC@Fe_3_O_4_) nanocomposites.

The proposed nanocomposite was fabricated via in situ oxidative free radical polymerization of acrylamide monomers within the vicinity of Cell@Fe_3_O_4_ nanoparticles [30]. Cell@Fe_3_O_4_ nanoparticles (8 g), acrylamide (6 g), and azobisisobutyronitrile (0.04 g) were introduced to a three-necked round-bottomed jar containing 100 mL of methanol, with an attractive bar and reflux condenser. The flagon was exposed to nitrogen for 20 min to maintain a vacuum environment and to remove any air pockets. This process was continued by mixing the blend at 60 °C for 6 h. After completion of the process, the blend was transferred into a Soxhlet device and deracinated using 60:40 DMF (dimethylformamide)/water for 72 h. The incorporated powdered material was then dried at 60 °C for 24 h in a vacuum oven. The complete synthesis and adsorption mechanism of the material is proposed in Figure 1.

### 2.2. Instrumentation

The surface morphology and fundamental composition of the nanocomposite were investigated using an electron amplifying instrument/energy-dispersive X-ray spectroscopy (SEM/EDX) (JSM, 6510LV, JEOL, Tokyo, Japan). The crucial dissemination and size of the nano support in the polymer network were characterized using TEM (JEM2100, JEOL, Tokyo, Japan). Inspection of the auxiliary functional groups present in the nanomaterial was facilitated using FTIR spectroscopy in the range of 400–4000 cm^−1^ using a Nicolet iS50 spectrometer (Madison, WI, USA) with KBr pellets. XRD characterization of the nanocomposite was done using a Bruker AXSD8 Advance X-beam diffractometer (Karlsruhe, Germany) with Cu Kα radiation (λ = 1.5406 Å) and a checking edge (10–80°). The textural properties of the nanocomposite were determined using N_2_ sorption–desorption isotherms, and the surface zone pore measurement was facilitated via the application of the Brunauer–Emmett–Teller (BET) and Barett–Joyner–Halenda (BJH) methods. Results were recorded at a fluid N_2_ temperature using a Nova Station instrument (NOVA, London, UK). The thermal stability of the integrated material was recorded using a Perkin Elmer STA 6000 analyzer (Perkin Elmer, Waltham, CA, USA) in a nitrogen environment at a warming rate of 10 °C/min. The variation of the surface charge for different surface pH conditions of the nanocomposite was analyzed by utilizing a zeta analyzer (Malvern Instruments Corporation, Malvern, UK).

### 2.3. Optimization Experiments

Adsorption tests were performed by utilizing the batch process for a fluid with an optimum pH and a temperature of 323 K. An amount of 0.03 g (1.5 g L^−1^) of nano-sorbent was added to 20 mL of metal particles with a concentration of 100 mg L^−1^, and the mixture was placed in an ultrasonicator at optimum conditions. Subsequently, the adsorbent was separated and the supernatant was collected for detection of Pb(II) concentration by atomic absorbance spectrophotometer (AAS, GBC Avanta M, Braeside, Australia). Analysis of the sonication time was performed over a period of 5–300 min using 100 mg L^−1^ of metal particles at an ideal pH. The impact of the adsorbent portion and the metal particles was also considered. Measurement of metal particles adsorption onto PAC@Fe_3_O_4_ was performed using the mass equalization relationship, as follows:

(1) $$q_{e}=\frac{(C_{0}-C_{e})V}{W}$$

where *q_e_* is the proportion of Pb(II) adsorbed per unit gram of the adsorbent (mg g^−1^); *C*_0_ and *C_e_* are the concentrations of the Pb(II) in the initial course of action (mg L^−1^) and due to sequestration; the proportion of the adsorbate solution is represented as *V* (0.02 L); *W* is the proportion of the nanomaterial used (g).

### 2.4. Isotherm Studies

The isotherm studies related to the adsorption process depict the effect of metal ion centers around the proportion of Pb(II) accumulated on the surface of the adsorbent to achieve the optimum parity position in the scavenging technique. In the current study, Langmuir [31], Freundlich [32], Temkin [33], and Redlich–Peterson [34] models were used with the test data.

(2) $$q_{e}=\frac{q_{m}K_{L}c_{e}}{1+K_{L}c_{e}}$$

(3) $$q_{e}=K_{F}{c_{e}}^{1/n}$$

(4) $$q_{e}=Bln(Ac_{e})$$

(5) $$B=\frac{RT}{b}$$

(6) $$q_{e}=\frac{K_{R}C_{e}}{1+a_{R}{C_{e}}^{g}}$$

The most prominent single layer adsorption capacity of the material is *q_m_* (mg g^−1^), where *K_L_* is the Langmuir constant (L mg^−1^) associated with the free energy of adsorption. The parameter *K_F_* (mg g^−1^) is the sorption capacity for Pb(II) according to the Freundlich model, while 1/*n* represents the adsorption constraint and is a marker for the favorability of adsorption. The estimation for *n* > 1 addresses extraordinary adsorption conditions. The parameter *R* is the gas constant, which has a value of 8.314 J mol^−1^ K^−1^; *T* is the preeminent temperature (K); *b* is the Temkin constant related to the heat of adsorption (J mol^−1^); and *A* is the binding constant (L g^−1^). The variables *K_R_* (L g^−1^) and *a_R_* are the Redlich–Peterson (R–P) constants, and g is R–P constant with values in the range of 0–1.

### 2.5. Models for Kinetic Studies

The evaluation of the components of the sequestration process is indispensable in the application of this approach. To examine the component that oversees the adsorption method, the dynamic data were filtrated using Lagergren pseudo-first-order [35], pseudo-second-order [36], and intra-particle diffusion [37]. The non-straight plots for the given models are represented by Equations (7)–(9):

(7) $$q_{t}=q_{e}(1-e^{-k_{1}t})$$

(8) $$q_{t}=\frac{k_{2}{q_{e}}^{2}t}{1+k_{2}q_{e}t}$$

(9) $$q_{t}=k_{\mathrm{int}}t^{1/2}+C$$

where *q_e_* (mg g^−1^) and *q_t_* (mg g^−1^) are the adsorption capacity of Pb(II) at equilibrium and time *t* (min), respectively, while *k*_1_ (min^−1^), *k*_2_ (g mg^−1^ min^−1^), and *k*_int_ are related to the rate constants for the pseudo-first-order model, pseudo-second-order model, and the intra-particle diffusion rate constant (mg g^−1^ min^½^).

### 2.6. Thermodynamic Aspect of Adsorption

Various thermodynamic parameters, such as entropy change, enthalpy change, and Gibbs energy, were evaluated to better understand the behavior of adsorption reaction with respect to temperature [38]. For this purpose, the thermodynamics of the process was investigated by using the Gibbs and van’t Hoff equations, represented by Equations (10)–(12):

(10) $$\Delta G^{o}=-RT\ln K_{c}$$

(11) $$\ln Kc=-\frac{\Delta H^{o}}{RT}+\frac{\Delta S^{o}}{R}$$

where *R* is the gas constant, *K_c_* (Cad/Ce) is the distribution coefficient, and *T* is the temperature of the course of action in degrees Celsius. The values of Δ*G°* and Δ*S°* were determined from the slope and intercept of a graph of ln*K_c_* as a function of 1/*T*, as shown in Figure S1. The free energy change (Δ*G°*) can be determined based on the provided optimized experimental conditions, such as optimum temperature range:

(12) $$\Delta G^{o}=\Delta H^{o}-T\Delta S^{o}$$

### 2.7. Non-Direct Chi-Square (χ^2^) Test

Non-linear relapses, such as the Chi-square (*χ*^2^) test, normally include the minimization or expansion of error conveyance between the test information and the anticipated isotherm, depending on the union criteria [39]. The non-linear chi-square (*χ*^2^) test is an investigative tool for error measurement and is vital for the best effective analysis of the adsorption information. A small value of *χ*^2^ indicates that the information associated with the isotherm is similar to that of the trial data, while a large value is indicative of the variety of the test information. The main advantage of utilizing the Chi-square test is that it facilitates the examination of all isotherms on a similar abscissa and ordinate.

(13) $$\chi^{2}=\frac{\sum_{i=1}^{n}{{\left(q_{e,cal}-q_{e,\exp}\right)}_{i}}^{2}}{q_{e,\exp}}$$

### 2.8. Desorption Experiments

The recuperation of PAC@Fe_3_O_4_ was attempted via the introduction of the Pb(II) adsorbed PAC@Fe_3_O_4_ with 0.1 M HNO_3_ solution for four successive cycles, and desorption was determined using Equation (14):

(14) $$\%Desorption=\frac{amount\;of\;metal\;ion\;desorbed\;to\;desorbing\;media}{amount\;of\;metal\;ion\;adsorbed\;on\;PAC@Fe_{3}O_{4}}\times 100$$

### 2.9. Definition of ANN

The ANN module of MATLAB’s statistical toolbox, R2013a, was utilized to predict the output of the adsorption process using a two-layer ANN, Levenberg–Marquardt back-propagation algorithm with 1000 iterations, a tangent sigmoid transfer function (tansig) at the hidden layer, and a linear transfer function (purelin) at the output layer. In this study, four neurons were applied at the initial Pb(II) concentration of 20–100 mg L^−1^), while adsorbent mass (0.5–5 g L^−1^), contact time (5–240 min), pH (1–6), one neuron (removal efficiency) in the output layer, and 1–20 neurons in the hidden layer were also applied. The normalization of the data was performed in the range of 0–1 to avoid the numerical imbalance due to small and large experimental weights [40,41]. The normalization equation is given by Equation (15):

(15) $$y=\frac{x_{i}-x_{\min}}{x_{\max}-x_{\min}}$$

where *y* belongs to the systematized value of *x_i_*, and the minimum and maximum values of *x_i_* correspond to *x*_min_ and *x*_max_, respectively. The mean square error (MSE) and regression coefficient (*R*^2^) were defined to check the performance of the ANN models and are given as follows [27,28]:

(16) $$MSE=\frac{1}{N}\sum_{i=1}^{N}\left(\left|y_{prd,i}-y_{\exp,i}\right|\right){}^{2}$$

(17) $$R^{2}=1-\frac{\sum_{i=1}^{N}\left(y_{prd,i}-y_{\exp,i}\right)}{\sum_{i=1}^{N}\left(y_{prd,i}-y_{m}\right)}$$

where *y*_*prd*__,_*_i_* is the predicted value of the ANN model, *y*_exp,*i*_ is the experimental value, *N* is the number of data, and *y_m_* is the average of the experimental values.

## 3. Results and Discussion

### 3.1. Characterization of the Material

**Figure 2:**
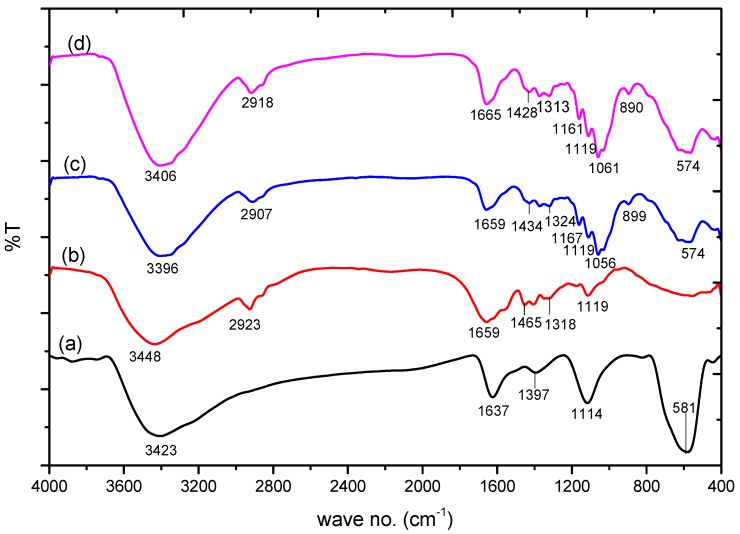
FTIR spectra of: (**a**) Fe_3_O_4_; (**b**) poly(acrylamide); (**c**) poly(acrylamide)-g-Cell@Fe_3_O_4_ (PAC@Fe_3_O_4_); and (**d**) PAC@Fe_3_O_4_-Pb.

Figure 2 shows the FTIR spectra of Fe_3_O_4_ nanoparticles (NPs) that depict a high-intensity band at 581 cm^−1^. This band is associated with the Fe–O stretching vibration from the magnetic nanoparticles [42], and the bands at 1637 and 3423 cm^−1^ are associated with the O–H stretching modes and bending vibration of water, respectively. In the FTIR spectra of PAM, the absorbance band at 3448 cm^−1^ is due to amidogen. The absorbance peaks at 2923 cm^−1^ and 1659–1119 cm^−1^ are associated with the –CH_2_ stretching vibrations, C=O stretching vibrations, N–H bending vibration, and C–N bond, respectively [43]. The FTIR spectra of PAC@Fe_3_O_4_ reveals that the band at 3396 cm^−1^ is due to the –OH stretching vibration of cellulose, PAM, and Fe_3_O_4_.

The other bands at 2915 cm^−1^ are attributed to the C–H asymmetric and symmetric stretching vibrations of the cellulosic ring and PAM. The peak at 1659 cm^−1^ is associated with the –C=O bond from PAM. The loop at 1324 cm^−1^ is a direct result of the OH bending vibrations. The absorption peaks at 1167–899 cm^−1^ are related to the –CH_2_ parallel scissoring in the cellulosic pyranoid ring, C–O anti-symmetric bridge stretching, the crystal absorption peak of the cellulose C–O–C pyranoid ring skeletal vibrations, and β-glycosidic linkages [44]. It was determined that the peak at 574 cm^−1^ is associated with the Fe–O bond. The FTIR spectra of PAC@Fe_3_O_4_ after adsorption of Pb(II) indicates a shift in the frequency at certain band points, suggesting the involvement of the –OH and –NH_2_ groups for adsorption in the encapsulation process.

Figure S2 represents the XRD spectra of cellulose and PAC@Fe_3_O_4_. The characteristic peaks of cellulose are present at 2θ values of 22.27° and 34.28° [45]. After uniting and fusing Fe_3_O_4_ MNPs in the matrix of PAM-g-Cell, the crystalline structure transitioned to a semi-crystalline structure and there appeared to be specific crests in the XRD spectra of PAC@Fe_3_O_4_ at 42.43°, 56.62°, and 62.38°. These are characteristic peaks of Fe_3_O_4_ MNPs.

**Figure 3:**
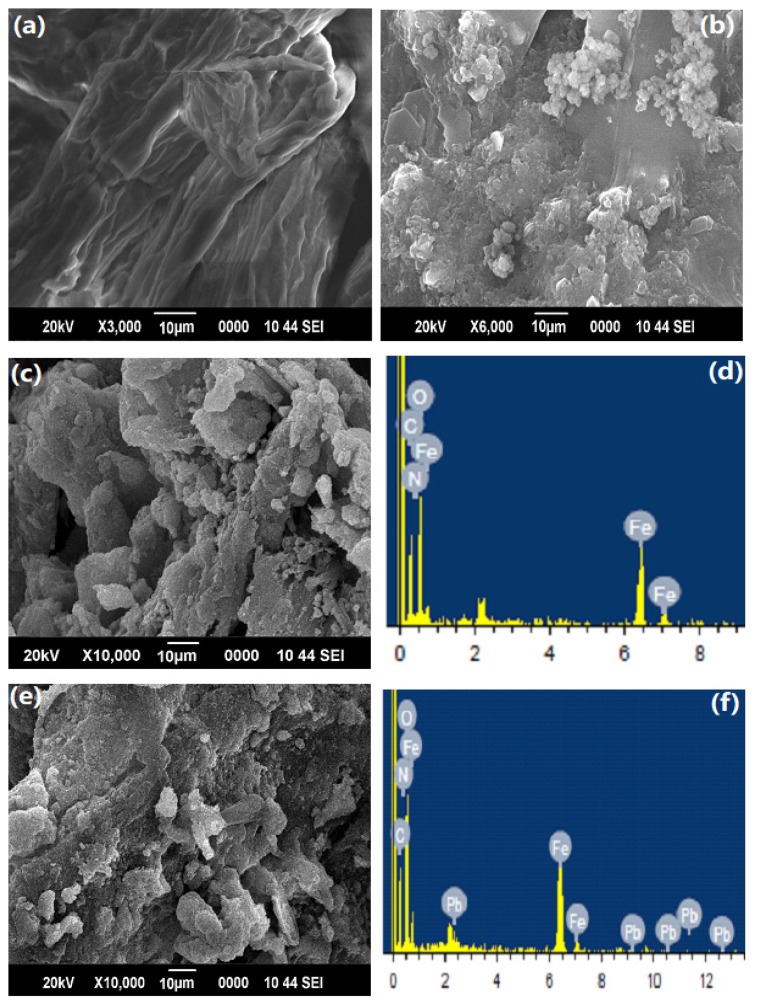
SEM images of: (**a**) cellulose; (**b**) PAM; (**c**,**d**) PAC@Fe_3_O_4_ and its EDX image before adsorption; and (**e**,**f**) PAC@Fe_3_O_4_ and its EDX image after adsorption of Pb(II).

The morphology of cellulose, PAM, and PAC@Fe_3_O_4_ nanocomposite, in addition to the EDX images before and after adsorption of Pb(II), are presented in Figure 3a–f. The structure of cellulose appears to be sinewy and smooth in the SEM image, while the PAM has an apparently flaky surface. Nonetheless, after fusion with PAM and support of the Fe_3_O_4_ MNPs, the sinewy structure transforms into a permeable material. After adsorption of Pb(II), the permeable structure transforms into irregular flakes because of adsorption of water molecules on the surface of the material, as shown in Figure 3e. The SEM images are the basis of the inference that cellulose was effectively united with PAM with the reinforcement of Fe_3_O_4_ MNPs nanoparticles in the hybrid copolymer framework.

EDX (Table 1) images of PAC@Fe_3_O_4_ before and after treatment were acquired to confirm the adherence of metal ions onto the exterior of PAC@Fe_3_O_4_, as shown in Figure 3d,f. The appearance of the Pb(II) bar in the EDX spectrum of Pb(II) adsorbed on PAC@Fe_3_O_4_ indicates that there is adherence between the Pb(II) ions and the surface of the material.

**Figure 4:**
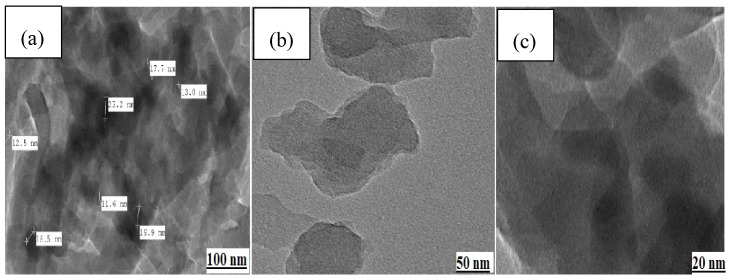
TEM images of PAC@Fe_3_O_4_ showing nanoparticle dispersion in the polymer matrix at (**a**) 100 nm, (**b**) 50 nm, and (**c**) 20 nm magnification ranges.

It is difficult to analyze the distribution of Fe_3_O_4_ MNPs in the polymer matrix because of the low magnification in the SEM micrographs. Thus, an appropriate technique for the analysis of the polymer framework is the utilization of TEM. Based on the TEM micrographs shown in Figure 4, Cell@Fe_3_O_4_ in association with PAM forms a nanocomposite, in which the nanoparticles are distributed throughout the polymer lattice. It is evident that the Fe_3_O_4_ MNPs were consistently covered by the PAM-g-Cell matrix. The average size of the Fe_3_O_4_ MNPs was 20.5 nm.

**Figure 5:**
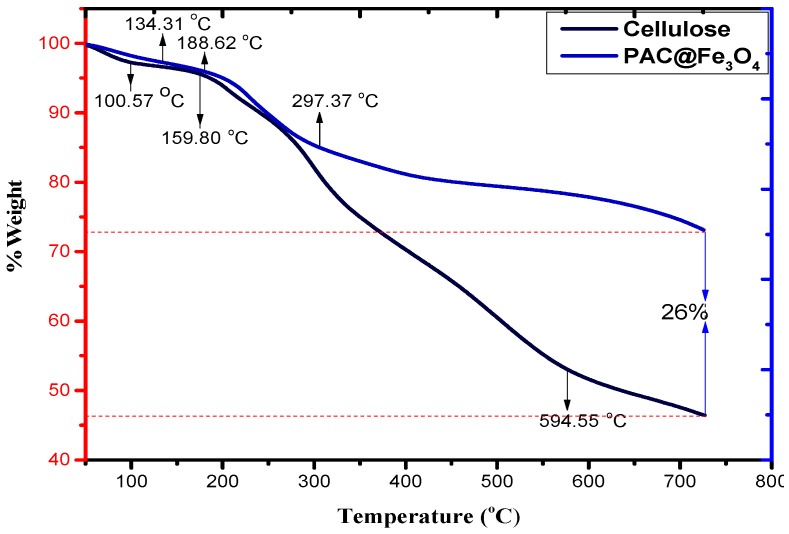
TGA spectra of cellulose and PAC@Fe_3_O_4_ in a temperature range of 0–800 °C.

Thermogravimetric techniques are effective strategies for illustrating the temperature response of a material to heat. Figure 5 shows the TGA spectra of cellulose and PAC@Fe_3_O_4_. From this figure, it is evident that the deterioration of pristine cellulose occurs in two stages. The initial step at 100.57 °C occurs because of the loss of hydrated and constituent water. The second stage involves weight reduction that occurs at approximately 159.80 to 594.55 °C. This is generally associated with the deterioration of cellulose (natural carbon) under oxidizing conditions [46]. Disintegration past 594.55 °C occurs because of oxidation of the decayed products of cellulose. In addition, PAC@Fe_3_O_4_ also undergoes a two-stage deterioration, as the first one indicated by a peak at 134.31 °C is due to the loss of water molecules. The second step occurs at approximately 188.62 to 297.37 °C, which can be inferred from cellulose deterioration. Weight reduction at more than 297.37 °C decreases because of the exceedingly stable spinel-organized Fe_3_O_4_ nanoparticles in the framework of the biopolymer. For the correlation of TGA thermograms of cellulose and PAC@Fe_3_O_4_ nanocomposites, it was determined that on average, 26% of the Fe_3_O_4_ nanoparticles are available in the polymer framework.

**Figure 6:**
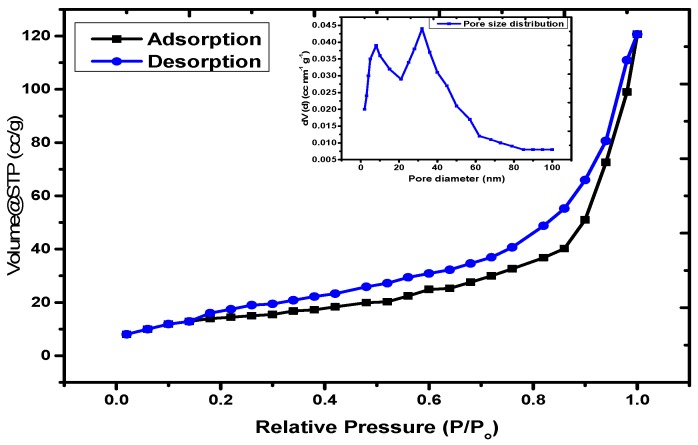
N_2_ adsorption–desorption curve and BJH pore size distribution for the PAC@Fe_3_O_4_ nanocomposite.

The surface area and porosity of the material can be evaluated using the N_2_ adsorption–desorption curve at low temperature. The assembled isotherm and pore size distribution controlled by using the BJH model is illustrated in Figure 6. The data reveals that the material can be considered as a mesoporous solid with a type IV isotherm based on to the IUPAC nomenclature, with pore closeness of various breadths [47]. From the literature, the surface territory and porosity of neat Fe_3_O_4_ nanoparticles were observed to be 286.9 m^2^/g and 0.6928 cm^3^/g, respectively [48]. The normal hysteresis circle for this case is type H1, which is related to the permeable materials that display a limited dispersion of moderately uniform round and hollow pores [49]. The BET surface region of the nanocomposite produced by the N_2_ adsorption–desorption technique was determined to be 65.89 m^2^/g, with a BJH porosity of 0.073 cm^3^/g. The surface zone and explicit pore volume of Fe_3_O_4_ diminished in the PAC@Fe_3_O_4_ nanocomposite because of the disappearance of the nanoporosity of Fe_3_O_4_ nanoparticles due to functionalization with the PAM-g-Cell polymer lattice.

**Figure 7:**
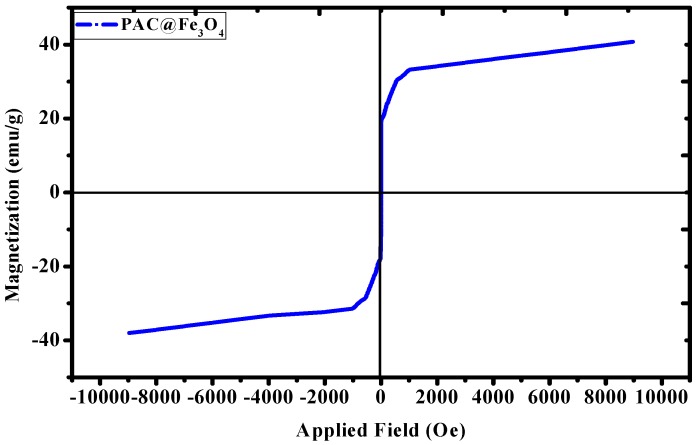
Magnetization curve for the PAC@Fe_3_O_4_ nanocomposite at room temperature.

The magnetic property of the material was analyzed by using a vibrating sample magnetometer (VSM) PAR 155 (Quantum Design, Manchester, UK) in the range of −10000 to +10000 Oe. The magnetic hysteresis (M–H) loop indicates that the material is showing ferromagnetic behavior at room temperature [50]. The saturation magnetization (*m_s_*) value of Fe_3_O_4_ MNPs depends upon the synthesis protocol and type of non-magnetic groups attached to their surfaces [51]. From the literature, it was found that the *m_s_* value for bare Fe_3_O_4_ MNPs was 56.8 emu g^−1^ [52], 75 emu g^−1^ [53], 71.2 emu g^−1^ [54], and 56 emu g^−1^ [55], based on the difference in synthesis protocol. While looking at the Figure 7, the *m_s_* value for the PAC@Fe_3_O_4_ was found to be 40.80 emu g^−1^. The decrement in the *m_s_* value of bare Fe_3_O_4_ magnetic nanoparticles (MNPs) suggests the functionalization of non-magnetic polyacrylamide-g-cellulose matrix on the surface. Thus, based on the VSM studies, it was concluded that there is a correlation between Fe_3_O_4_ MNPs and the polymer matrix, with a negative synergistic effect on magnetic properties due to the presence of non-magnetic mass.

Figure S3 shows the zeta potential curve for PAC@Fe_3_O_4_ as a function of pH, using 0.1 M KCl solution for a pH range of 1–10. Examination of this result revealed that the isoelectric point (IEP) of the material had a pH of 4.32. Hence, the surface of the nano-sorbent is positive below this pH value and negative above this value. The maximum and minimum values of the zeta potential were determined to be +35.68 mV at pH 1 and –38.67 at pH 10.

### 3.2. Adsorption Behavior of PAC@Fe_3_O_4_ towards a Ternary System

#### 3.2.1. Impact of pH

**Figure 8:**
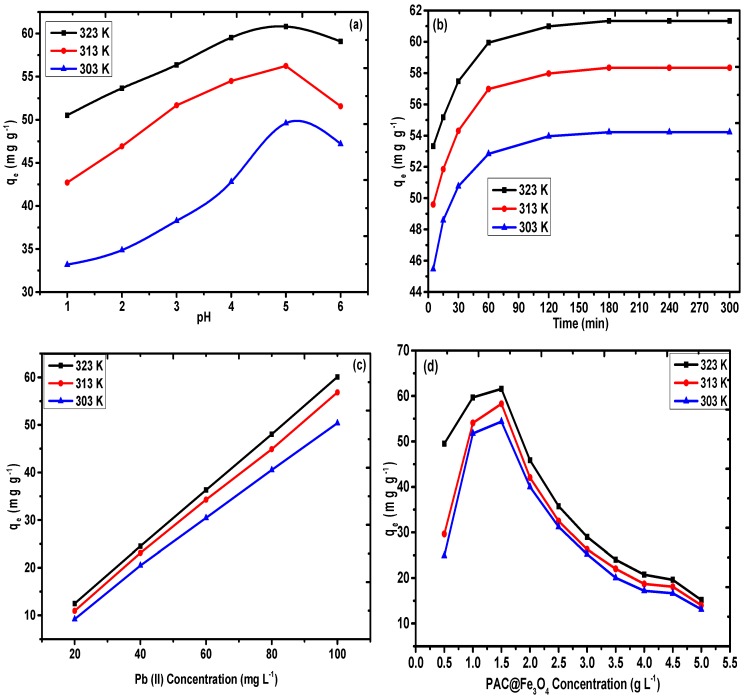
Effect of (**a**) pH, (**b**) agitation time, (**c**) Pb(II) concentration, and (**d**) adsorbent dose on adsorption of Pb(II) on PAC@Fe_3_O_4._

The pH of the process is the elementary variable in the sequestration response and the impact of this parameter was analyzed for values in the range of 1–7 with a precision of ±0.1, by keeping the other variables constant in the temperature range of 303–323 K. Figure 8a depicts the impact of pH on the sequestration rate of Pb(II) on PAC@Fe_3_O_4_. The sequestration rate of Pb(II) increased with the increase of the pH to a maximum value of pH 5 for Pb(II), because of the protonation of hydroxyl and carboxyl functional groups on the exterior of PAC@Fe_3_O_4_. Contrary to the experimental results, beyond pH 5, the take-up rate will decrease overall because of the complexation of metal ions as hydroxide. Therefore, the pH of 5 was selected as the ideal pH for Pb(II) for further examinations, because the best adsorption efficiencies of 60.82 mg g^−1^ at 323 K, 56.24 mg g^−1^ at 313 K, and 49.6 mg g^−1^ at 303 K were observed at this pH.

#### 3.2.2. Impact of Contact Time

The agitation time is a pivotal variable that represents the impact of the adsorption. To determine the ideal time for maximum adsorption, a batch technique was performed that utilized 1.5 g L^−1^ of PAC@Fe_3_O_4_ in Pb(II), with a concentration of 100 mg L^−1^ in a temporal range of 5–300 min and a temperature range of 303–323 K. The extent of the accumulated Pb(II) was controlled using a mass balance equation, represented by Equation (1). This behavior can be presented as a graph of agitation time vs. adsorption capacity, as shown in Figure 8b. The sequestration of metal ions is highly energetic in the initial 180 min, due to the availability of active functional sites on the surface of the adsorbent. In the following stage, with the consistent decline in the number of active sites, the take-up of Pb(II) is moderate, and no further change in the take-up phenomenon was observed after 180 min. The adsorption limit was determined to be 61.34 mg g^−1^ at 323 K, 58.34 mg g^−1^ at 313 K, and 54.22 mg g^−1^ at 303 K, for an agitation time of 180 min. This duration was selected as the optimum time for the adsorption of Pb(II).

#### 3.2.3. Impact of the Initial Metal Ion Concentration

The initial metal ion concentration is an important driving force to overcome the mass transfer resistance of metal ions between the aqueous and solid phases [56]. A set of initial metal ion concentrations ranging from 20–100 mg L^−1^ were selected to observe the variation of adsorption capacity of PAC@Fe_3_O_4_ with respect to the bulk metal ion concentration. The impact of metal ion concentration is represented in Figure 8c. It was observed that increasing the metal ion concentration resulted in a simultaneous increase in the adsorption efficiency of the material. This indicates that there was an increasing number of collisional interactions between the surface-active groups and the reactive metal ions [42]. The highest adsorption capacity with 100 mg L^−1^ Pb(II) was discovered as 61.34 mg g^−1^ at 323 K, 58.34 mg g^−1^ at 313 K, and 54.22 mg g^−1^ at 323 K.

#### 3.2.4. Impact of Adsorbent Dose

One of the important batch parameters that greatly effects the behavior of the scavenging process is the amount of material used during the reaction. An amount in the range of 0.5 to 5 g L^−1^ was added to 100 mg L^−1^ of Pb(II) solution for 180 min, and the variation of the concentration of adsorbent on the removal rate of Pb(II) and the outcomes are presented in Figure 8d. It appears that the variation of the adsorbent concentration towards higher values is indicative of an increase in the removal rate of Pb(II) up to 1.5 g L^−1^ (0.03 g). However, further expansion of the concentration of the adsorbent reduces the removal rate. This pattern can be elucidated as the concentration of the adsorbent expands.

This pattern facilitates an increment in the number of particles in the solution, thereby providing a high density of surface-active sites for scavenging processes. However, further increments of the concentration of the adsorbent removal rate are minimized because of partial aggregation of adsorbent particles. As such, 1.5 g L^−1^ (0.03 g) was established as an ideal adsorbent concentration for further experimentation.

#### 3.2.5. Impact of Temperature

The impact of temperature on the sequestration of Pb(II) can be observed in Figure 8a–d in the temperature range of 303–323 K. The variation of temperature in increasing order (i.e., from 303–323 K) accelerates the reaction kinetics due to the increase of the diffusion rate of metal ions across the external boundary layer and within the pores of the PAC@Fe_3_O_4_ nanocomposite. In addition, at the maximum temperature, the energy of the system also facilitated the binding of Pb(II) on the surface of PAC@Fe_3_O_4_, thereby indicating that the sequestration of Pb(II) is controlled by an endothermic process. As a result, 323 K was selected as the ideal temperature for the remaining experiments.

### 3.3. Adsorption Isotherms

**Figure 9:**
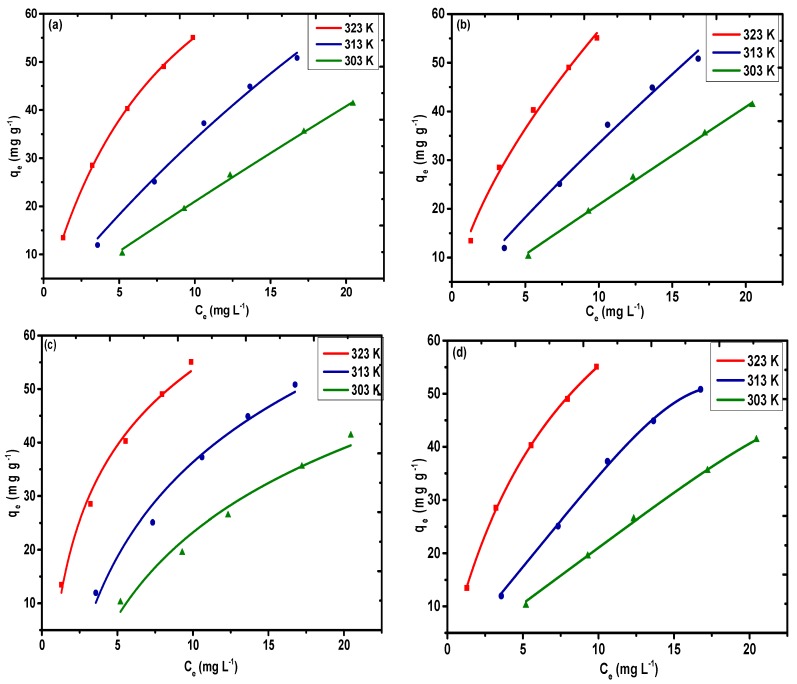
Adsorption isotherms for (**a**) Langmuir, (**b**) Freundlich, (**c**) Temkin, and (**d**) Redlich–Peterson models for Pb(II) on PAC@Fe_3_O_4_ at 323, 313, and 303 K (dose = 1.5 g L^−1^ and pH 5).

According to the Langmuir model, a reversible chemical equilibrium exists between the surface of the material and the bulk solution, which is only limited for a finite number of active sites. The non-linear plot (Figure 9a) for the Langmuir model was selected for Pb(II) based on non-linear regression. It can be observed from Table 2 that the highest monolayer adsorption capacities (*q_m_*) controlled by the Langmuir model are 314.47, 239.34, and 100.79 mg g^−1^ for Pb(II) at 323, 313, and 303 K, respectively.

According to the Freundlich model, adsorption on the surface of a solid always follows the principle of heterogeneity (i.e., the formation of a multilayer of guest molecules on the surface). The values of K_F_ and *n* were obtained by applying the equilibrium data to the non-linear regression (Figure 9b). The interpretation of the type of interactions that occur between the metal ions and the sorbent is possible based on the value of the Freundlich constant *n*. If the value of *n* is greater than 1, then there will be strong interactions between Pb(II) and the sorbent (i.e., favorable adsorption). However, if *n* is less than 1, this does not favor adsorption. It is evident from Table 2 that for all the temperature runs, the estimation of *n* is higher than 1, which indicates that the adsorption of Pb(II) is favorable under the given optimized conditions. The values of *R*^2^ and *χ*^2^ were determined to be (0.99, 2.59) at 323 K, (0.98, 3.69) at 313 K, and (0.98, 0.57) at 303 K. The value of K_F_ was determined to be 13.14 mg g^−1^(dm^3^/g)*^n^* at 323 K, 4.48 mg g^−1^(dm^3^/g)*^n^* at 313 K, and 2.22 mg g^−1^(dm^3^/g)*^n^* at 303 K.

Temkin isotherms take into account the impact of the heat of adsorption, which diminishes with the interactions of the adsorbate and the adsorbent. The non-linear plot (Figure 9c) of *q_e_* versus *C_e_* facilitates the selection of the values of A_T_ and b_T_. The Temkin constants incorporated in Table 2 clearly suggest that the adsorption proceeds via chemisorption of the Pb(II) rather than the ion exchange mechanism. The values for the binding constant A_T_, given in Table 2 as 1.39, 0.42 L, and 0.28 L mg^−1^, account for the high proclivity of Pb(II) towards the adsorbent surface at 323, 313, and 303 K. The b_T_ values are 131.96 J mol^−1^ at 323 K, 105.32 J mol^−1^ at 313 K, and 118.23 J mol^−1^ at 303 K. The *R*^2^ and *χ*^2^ values were determined to be (0.98, 3.72) at 323 K, (0.97, 5.64) at 313 K, and (0.97, 4.53) at 303 K. The value of g (0.96, 5.31, and 5.81) calculated using the R–P model (Figure 9d) correspond to unity, indicating that the Langmuir model is the best model for analysis of the equilibrium data. Therefore, based on the higher value of the regression coefficient and the lower value of *χ*^2^ (i.e., for Pb(II)), the values are 0.99, 0.99, and 0.99 and 0.10, 0.31, and 0.47 at 323, 313, and 303 K, respectively. This indicates that the Langmuir model is the best fitting model for fundamental experimental information in all temperature ranges.

### 3.4. Adsorption Kinetics

**Figure 10:**
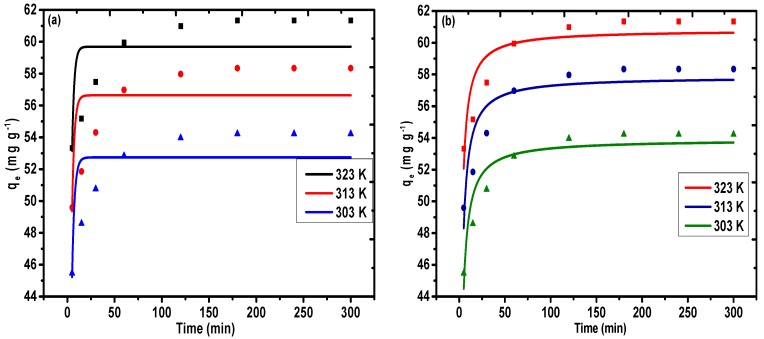

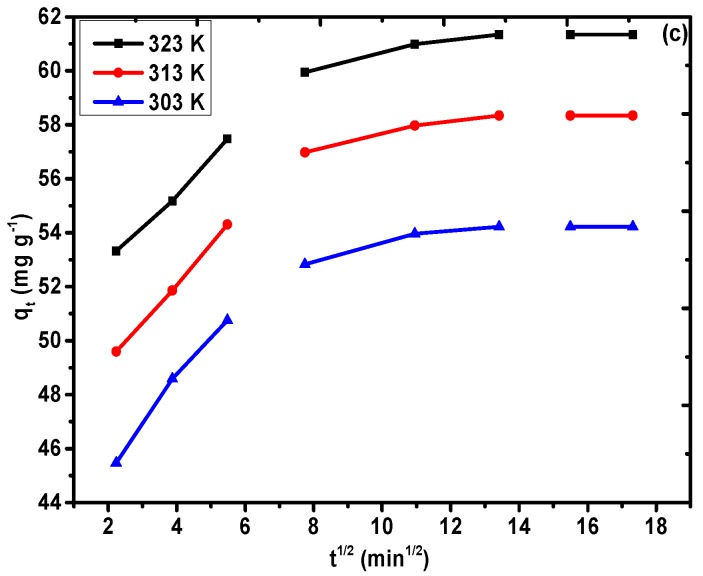
Non-linear regression plot of the (**a**) pseudo-first-order model, (**b**) pseudo-second-order model, and (**c**) intra-particle diffusion plot for Pb(II) on PAC@Fe_3_O_4_ at 323, 313, and 303 K (dose = 0.03 g and pH 5).

Figure 10a–c was utilized to fit the equilibrium information obtained from the adsorption of Pb(II) (100 mg L^−1^) on PAC@Fe_3_O_4_ at an ideal pH and at temperatures of 323, 313, and 303 K. The values of the dynamic variables *R*^2^ and *χ*^2^, which were determined using the non-linear method based on dynamic equations, are recorded in Table 3. It is evident that the *R*^2^ values (0.79, 0.82, and 0.89) are sufficiently large in combination with the low values of *χ*^2^ (2.04, 2.06, and 1.17) for the application of the pseudo-second-order model (Figure 9b), followed by the pseudo-first-order model (*R*^2^ = 0.43, 0.46, and 0.56; *χ*^2^ = 5.75, 6.30, and 4.67) (Figure 9a) at 323, 313, and 303 K. In addition, the *q_e,cal_* values (60.80, 57.85, and 53.89 mg g^−1^) selected from the pseudo-second interest condition were determined to potentially concur with the *q_e,exp_* values (61.34, 58.34, and 54.22 mg g^−1^). The *q_e,cal_* values (59.69, 56.64, and 52.74 mg g^−1^) evaluated from pseudo-first-order dynamic conditions have a base synchronization with the fundamental values, *q_e,exp_* (61.34, 58.34, and 54.22 mg g^−1^). Therefore, the adsorption framework for Pb(II) onto PAC@Fe_3_O_4_ can be best depicted as a pseudo-second-order pathway, and the rate-determining step is possibly chemisorption at 323, 313, and 303 K. The higher estimation of k_2_ of 0.019 g mg^−1^ min^−1^ at 323 K, 0.017 g mg^−1^ min^−1^ at 313 K, and 0.016 g mg^−1^ min^−1^ at 303 K indicates higher transport of Pb(II) from bulk to PAC@Fe_3_O_4_ surfaces at a high temperature. Figure 9c demonstrates the intra-particle diffusion model, suggesting that the sequestration process is facilitated by a diffusion mechanism.

### 3.5. Adsorption Thermodynamics

The thermodynamic parameters related to Pb(II) sequestration by the synthesized material were calculated using the Gibbs equation, and a plot of ln*K_c_* vs 1/*T* is given in Figure S1. The positive values of Δ*H°* of 4.86 KJ mol^−1^ at 303 K, 5.66 KJ mol^−1^ at 313 K, and 7.30 KJ mol^−1^ at 323 K indicate that the adsorption process of Pb(II) on PAC@Fe_3_O_4_ is endothermic. The values of Δ*G°* were negative for expansion in the range of 303–323 K. The negative magnitude of Δ*G°* increased with expansion in temperature range, suggesting that Pb(II) adsorption on the nanocomposite surface is unconstrained and the speed increases with temperature [57]. The positive estimation of Δ*S°* (0.031 KJ mol^−1^ K^−1^ at 303 K, 0.033 KJ mol^−1^K^−1^ at 313 K, and 0.038 KJ mol^−1^ K^−1^ at 303 K) reveals intercession behavior and the increase of the degrees of freedom at the adsorbent–approach interface, with the confinement of the metal ions to the active sites of the adsorbent. This is indicative of the partial release of the solvated metal ion from the solvent molecule prior to adsorption, facilitating random behavior in the system.

### 3.6. Adsorption Mechanism

Different mechanisms, such as the electron donor–acceptor complex, π–π interactions, and solvent impact, have been proposed to elucidate the adsorption mechanism of metal ions on the surface of nanocomposite materials [58]. Based on the FTIR spectra shown in Figure 2d, a metal donor–acceptor complex formation mechanism can be considered. In this case, the dynamic –NH_2_, –OH groups on the surface of the nanocomposite act as electron donors and the metal ions behave as electron acceptors. Examination of the EDX results shown in Figure 3d,f, reveals that after adsorption of Pb(II), the N top completely vanishes from the spectra. This indicates that alongside the –OH groups, –NH_2_ groups take part in a complex formation with Pb(II). The kinetic variables in Table 3 demonstrate that the process of removal is controlled by chemical adsorption and may undergo electron donor–acceptor complex formation, in which a complex is formed between Pb(II) and PAC@Fe_3_O_4_ active surface sites.

### 3.7. Impact of Other Competitive Ions

**Figure 11:**
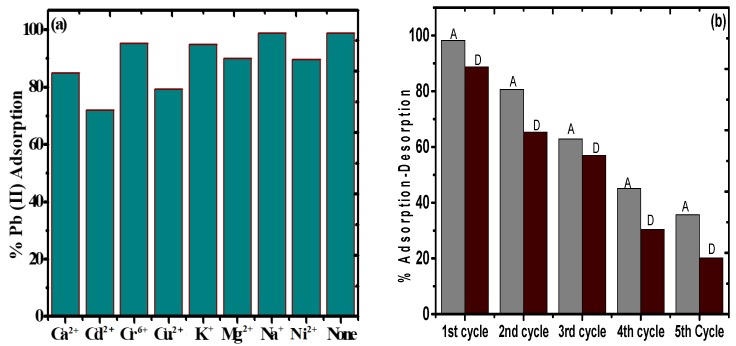
(**a**) Effect of other competitive ions on the adsorption of Pb(II) by PAC@Fe_3_O_4_. (**b**) Adsorption–desorption plot for Pb(II) on PAC@Fe_3_O_4._

Figure 11a depicts the impact of various metal ions including Na^+^, K^+^, Mg^2+^, Ca^2+^, Cu^2+^, Cd^2+^, Ni^2+^, and Cr^6+^ on the % adsorption of Pb(II) by PAC@Fe_3_O_4_. It was discovered that in the absence of other co-ions, the adsorption of Pb(II) was approximately 99%. For 20 mL of 50 mg L^−1^ solution of NaNO_3_, the adsorption effectiveness of Pb(II) was observed to be 99%, indicating that the proximity of the sodium particles does not have a synergistic impact on Pb(II) adsorption. The proximity of 20 mL of 50 mg L^−1^ solution of KNO_3_ caused a decrease of the adsorption proficiency to 96% for Pb(II), demonstrating a less significant effect of K^+^ ions on Pb(II) adsorption. The presence of Mg^2+^ ions significantly impacts the adsorption of Pb(II) because of identical charge valence for a specific adsorption site on the surface of the material. The mg^2+^ particles significantly reduce the adsorption capacity of Pb(II) from 99% to 90%. With a larger size and competing valency, Ca^2+^ reduces the efficiency of the material towards Pb(II) from 99% to 85%. The presence of Cr^6+^ ions show a very small effect on adsorption of Pb(II) by asserting a decline of only 4% on Pb(II) adsorption. This may be due to the application of high pH (5) condition, as the optimized pH for Cr^6+^ adsorption lies in the range of 2.5–3.5 [59], while the presence of Ni^2+^, Cu^2+^, and Cd^2+^ show a minimal to significant effect on Pb(II) adsorption, with decreased values of 90%, 79%, and 70% from 99%. Again, the solution pH plays an important key role here, as the optimum pH for Ni^2+^ adsorption is in the range of 6–7 [60], while for Cu^2+^ it is 5.5–6.5 and for Cd^2+^ it is 6–8 [61,62]. Therefore, it was concluded that the presence of Ca^2+^, Cu^2+^, and Cd^2+^ in the wastewater can significantly affect the Pb(II) on PAC@Fe_3_O_4_ based on the pH condition of the system.

### 3.8. Desorption and Regeneration Tests

The strength and reusability of an adsorbent are critical from an environmental and economic perspective. HNO_3_ (0.1 M) was utilized as a desorbing agent instead of HCl and Ethylenediaminetetraacetic acid (EDTA). PAC@Fe_3_O_4_ was utilized for 5 progressive adsorption–recovery cycles, and the results are presented in Figure 11b. Considering the adsorption–desorption process limit with regards to Pb(II), there was a reduction from 98% to 20.15% for Pb(II). Based on these outcomes, it was assumed that the PAC@Fe_3_O_4_ material can be utilized for multiple cycles.

### 3.9. Optimization of the ANN Structure

**Figure 12:**
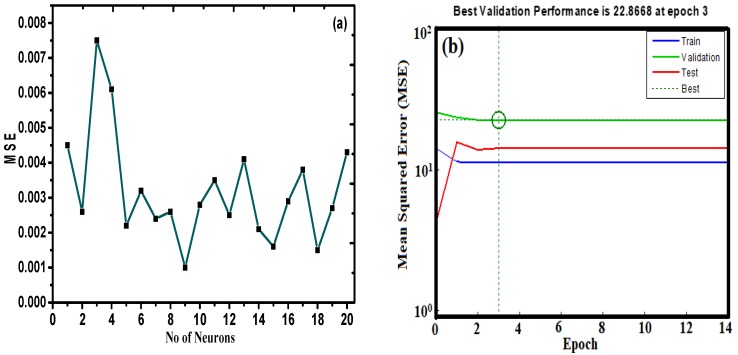
(**a**) Variation of number of neurons vs. mean square error (MSE) values. (**b**) MSE versus the number epochs for Pb(II) adsorption.

To train and test the neural network model, the ANN tool was utilized to compute the scavenging process based on the application of the experimental data at different operating conditions [40]. The optimization of the module was assessed with the aim of minimizing the MSE value and maximizing the *R*^2^ value of the testing set (1–20 neurons correspond to the hidden layer). Figure 12a illustrates the relationship between the neuron number of the hidden layer and the MSE for the Levenberg–Marquardt algorithm for Pb(II) adsorption. It is evident from the figure that with the increase in the number of neurons in the hidden layer, there is a decrease in value of MSE. Table 4 represents the variation of the number of neurons with respect to the *R*^2^ and MSE values for the ANN model. The results suggest that the *R*^2^ and MSE values are 0.9915 and 0.0010, respectively. As such, the model associated with 10 hidden neurons for the selected ANN module was preferred for the interpretation of the adsorption behavior of Pb(II) on the nano-sorbent.

Figure 12b represents MSE versus the number of the epoch curve. The results indicate that there was not a significant change in the performance of the method after epoch 3 for the proposed ANN module.

**Figure 13:**
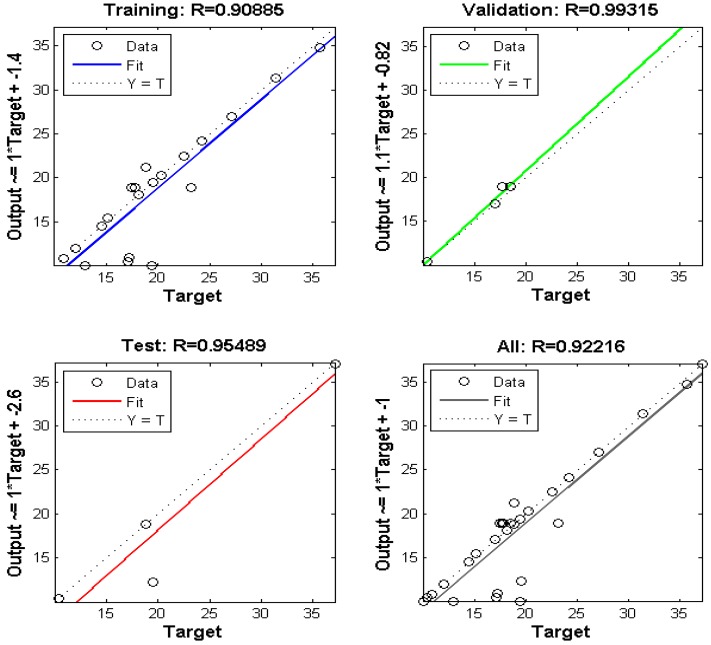
The experimental data versus the predicted data of normalized removal from the ANN for Pb(II).

As seen in Figure 13, there is a good agreement between the experimental data and the predicted data, which represents the predicted removal data for training and testing. The input and output data can be correlated to each other using an equation called the objective function, which is given as follows:

(18) ANNOutput=Purelin(w2∗tansig(w1∗[x(1);x(2);x(3);x(4)]+b1)+b2)

where *x*(1), *x*(2), and *x*(3) represent the inputs, *w*1 and *b*1 are the weight and bias of the hidden layer, respectively, and *w*2 and *b*2 are the weight and bias of the output layers, respectively.

Table 5 shows the weight and bias values of each layer, which were determined from the optimum ANN structure. Figure 13 represents the regression coefficient plot for the proposed ANN model. A value of 0.92 indicates a good correlation between the experimental and predicted data. The point data prediction by the ANN module was approximately equal to the values observed by the batch model (i.e., optimum time of 178.85 min, pH 4.89, adsorbent dose of 1.41 g L^−1^, and Pb(II) concentration of 98.55 mg L^−1^.

## 4. Conclusions

Fe_3_O_4_ NPs were synthetically changed by cellulose via copolymerization with poly(acrylamide) by free radical polymerization to create a novel, eco-friendly nano-sorbent (20.5 ± 1.5 nm), PAC@Fe_3_O_4_, for economical and proficient evacuation of Pb(II) (*q*_max_ = 314.47 mg g^−1^ at 323 K, 239.34 mg g^−1^ at 313 K, and 100.79 mg g^−1^ at 303 K) from a watery framework media at a pH of 5. The synthetic structure, surface properties, molecule size, and morphology of PAC@Fe_3_O_4_ were determined using fitting strategies. The fitting of the test data to various isotherm models under streamlined conditions confirmed the ability of the Langmuir model to elucidate the adsorption and the pseudo-second-order model to describe the adsorption rate condition. Thermodynamic examination depicts the adsorption as an endothermic and plausible physical process. It was possible to discharge the adsorbed metal particles from PAC@Fe_3_O_4_ using 0.1 M HNO_3_ to achieve a recuperation rate in excess of 35% for Pb(II) after five adsorption–desorption cycles. The incorporated nano-sorbent was confirmed to be a proficient and powerful stage-extractor of Pb(II) from wastewater and is potentially more efficient for extraction processes at the industrial level. As we know, most of the chemical industries discharge effluent, which contains Pb(II) in the range of 10–30 mg L^−1^. In the present study, the PAC@Fe_3_O_4_ material can remove 100 mg of L^−1^ Pb(II) with optimal efficiency and affinity. Thus, the present study has practical significance for wastewater treatment at the pilot scale in industries.

## Figures and Tables

**Table 1:** EDX analysis and percent composition of elements in PAC@Fe_3_O_4._

Elements	PAC@Fe_3_O_4_		PAC@Fe_3_O_4_-Pb	
	Weight %	Atomic %	Weight %	Atomic %
C	32.07	45.64	25.78	43.28
N	3.44	4.20	0.00	0.00
O	39.92	42.64	34.63	43.64
Fe	24.57	7.52	34.97	12.62
Pb	0.00	0.00	4.61	0.45

**Table 2:** Isotherm parameters for Pb(II) removal by PAC@Fe_3_O_4_ at 323, 313, and 303 K.

Model	Parameters	Pb (II)		
**Langmuir**		**323 K**	**313 K**	**303 K**
	*q_m_* (mg g^−1^)	314.47	239.34	100.79
	K_L_ (L mg^−1^)	0.121	0.016	0.003
	*R* ^2^	0.99	0.99	0.99
	*χ* ^2^	0.10	0.31	0.47
**Freundlich**	n	1.58	1.14	1.02
	K_F_ (mg g^−1^) (dm^3^/g)*^n^*	13.14	4.48	2.22
	*R* ^2^	0.99	0.98	0.98
	*χ* ^2^	2.59	3.69	0.57
**Temkin**	A_T_ (L mg^−1^)	1.39	0.42	0.28
	b_T_ (J mol^−1^)	131.96	105.32	118.23
	*R* ^2^	0.98	0.97	0.97
	*χ* ^2^	3.72	5.64	4.53
**Redlich‒Peterson**	g	0.96	5.31	5.81
	K_R_ (L g^−1^)	12.50	3.48	2.10
	a_R_ (L mg^−1^)	0.14	4.80	1.02
	*R* ^2^	0.99	0.99	0.99
	*χ* ^2^	0.14	0.58	0.44

**Table 3:** Kinetic parameters for Pb(II) removal by PAC@Fe_3_O_4_ at 323, 313, and 303 K, obtained through non-linear regression analysis.

Model	Parameters	Pb(II)		
Pseudo-First-Order		**323 K**	**313 K**	**303 K**
	qe (exp) (mg g^−1^)	61.34	58.34	54.22
	qe (cal) (mg g^−1^)	59.69	56.64	52.74
	k_1_ (min^−1^)	0.44	0.41	0.38
	*R* ^2^	0.43	0.46	0.56
	*χ* ^2^	5.75	6.30	4.67
Pseudo-Second-Order	qe (exp) (mg g^−1^)	61.34	58.34	54.22
	qe (cal) (mg g^−1^)	60.80	57.85	53.89
	k_2_ (g mg^−1^ min^−1^)	0.02	0.02	0.02
	*R* ^2^	0.79	0.82	0.89
	*χ* ^2^	2.04	2.06	1.17
Intra-particle Diffusion	Kid	0.51	0.53	0.53
	C	53.96	50.47	46.85
	*R* ^2^	0.78	0.76	0.73
	*χ* ^2^	1.09	1.21	1.23

**Table 4:** Comparison of the removal efficiency of 20 neurons in the hidden layer by the artificial neural network (ANN) model developed with the Levenberg–Marquardt algorithm for Pb(II) adsorption.

No. of Neurons	*R*^2^ Value	MSE Value
1	0.9241	0.0045
2	0.9572	0.0026
3	0.8901	0.0075
4	0.9323	0.0061
5	0.9845	0.0022
6	0.9744	0.0032
7	0.9648	0.0024
8	0.9778	0.0026
9	0.9799	0.001
10	0.9915	0.0028
11	0.9241	0.0035
12	0.9543	0.0025
13	0.9727	0.0041
14	0.9815	0.0021
15	0.9450	0.0016
16	0.9855	0.0029
17	0.9712	0.0038
18	0.9487	0.0015
19	0.9876	0.0027
20	0.9267	0.0043

**Table 5:** The weight and bias of the trained ANN for predicting the removal of Pb(II).

*W*1	*W*2	*b*1	*b*2
	[−0.16147 0.85147 −1.1877 1.125 1.8233 0.12975 −0.72917 1.6183 −0.068404 −0.31243 1.8263 1.0474 0.33743 1.7101 −0.165 1.2238 0.85285 −0.41348 0.57248 −0.15122]	[5.1699;	[−2.7344]
		3.2342;	
		2.4921;	
[2.3555 0.22624 1.4495 1.7728;		3.3494;	
−1.7946 1.2629 1.8741 1.1642;		−1.7066;	
1.6833 1.0471 0.20408 1.9717;		−2.0543;	
0.87665 1.9143 −2.0152 −0.58027;		−0.9055;	
0.21074 −0.44249 3.3956 −0.4547;		1.3696;	
0.059627 −1.0689 −0.5686 0.8507;		−0.92683;	
−1.6308 1.5808 2.2064 0.30565;		0.6882;	
1.8589 0.58103 −2.6895 −1.0942;		1.5333;	
−2.1314 −1.6081 −1.0178 −1.4578;		−3.3323;	
−2.9458 2.3503 −1.3511 0.86399;		−0.30565;	
0.63077 −1.5321 1.3034 −0.23255;		0.36439;	
1.3319 0.64905 −0.51145 2.138;		−1.6027;	
1.3292 0.083706 −1.8689 1.8066;		−0.30928;	
1.3137 −0.96565 1.7822 −3.1317]		3.1452;	
		2.623;	
		2.9375;	
		2.176]	

## Supplementary Materials

The following are available online at https://www.mdpi.com/2079-4991/9/12/1687/s1, Figure S1: Thermodynamic plot; Figure S2: XRD spectra; Figure S3: Zeta potential curve.

## Abbreviations

TGA	thermogravimetric analysis
FTIR	Fourier transform-infrared
XRD	X-ray diffraction
SEM	scattering electron microscopy
TEM	transmission electron microscopy
BET	Brunauer–Emmett–Teller
MNPs	magnetic oxide nanoparticles
PAM	poly(acrylamide)
EDX	energy-dispersive X-ray
ANN	artificial neural network
BJH	Barett–Joyner–Halenda
MSE	mean square error
