# Supplementary material for: In Situ Copolymerized Polyacrylamide Cellulose Supported Fe3O4 Magnetic Nanocomposites for Adsorptive Removal of Pb(II): Artificial Neural Network Modeling and Experimental Studies
Source: Nanomaterials (Basel). 2019 Nov 25;9(12):1687. doi: 10.3390/nano9121687 (PMC6955854; doi:10.3390/nano9121687)
Supplement: Supplementary file 1 [file nanomaterials-09-01687-s001.pdf]

Supporting Information

In Situ Copolymerized Polyacrylamide Cellulose Supported Fe_3O_4 Magnetic Nanocomposites for Adsorptive Removal of Pb(II): Artificial Neural Network Modeling and Experimental Studies

Imran Hasan [1,*], Rais Ahmad Khan [2,*], Walaa Alharbi [3], Khadijah H. Alharbi [4] and Ali Alsalme [2]

1 The Environmental Research Laboratory, Department of Chemistry, Chandigarh University, Mohali 140301, India

2 Department of Chemistry, College of Science, King Saud University, Riyadh 11451, Saudi Arabia; aalsalme@KSU.EDU.SA

3 Department of Chemistry, Faculty of Science, King Khalid University, P.O. Box 9004 Abha, Saudi Arabia; Wal-harbe@kku.edu.sa

4 Department of Chemistry, Science and Arts College, Rabigh Campus, King Abdulaziz University, Jeddah 21911, Saudi Arabia; khalharbe@kau.edu.sa

* Correspondence: imran.chemistry@cumail.in (I.H.); krais@ksu.edu.sa (R.A.K.);
Tel: +91-8171878193 (I.H.); +966-536745404 (R.A.K.)

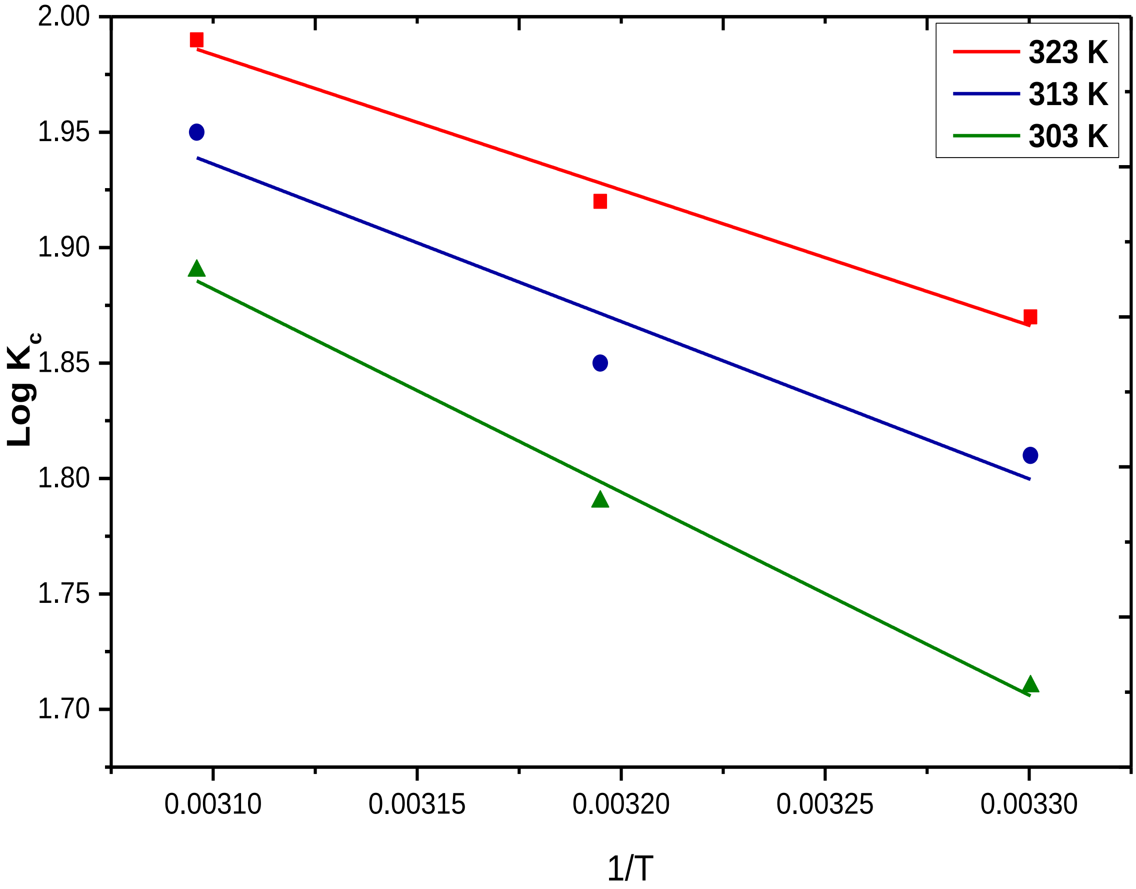

Figure S1. Thermodynamic plot for removal of Pb(II) on PAC@Fe_3O_4 at 323, 313 and 303 K.

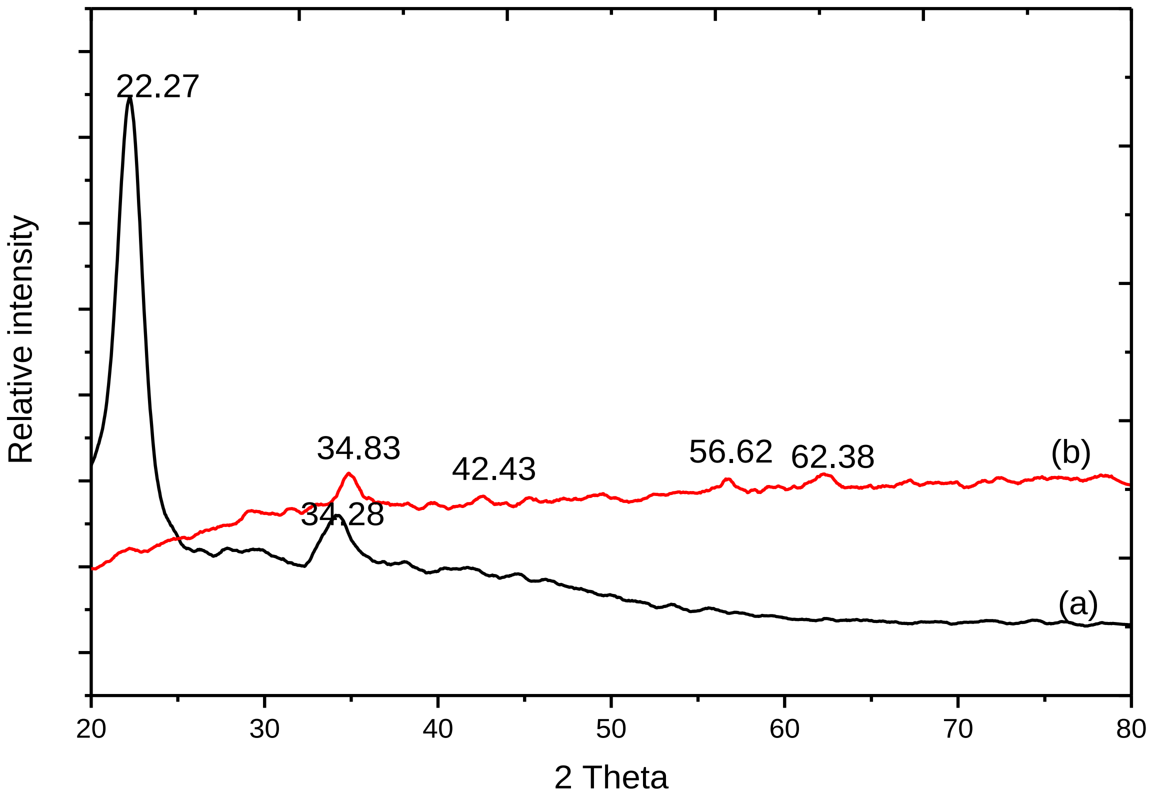

Figure S2. XRD spectra of (**a**) Cellulose and (**b**) PAC@Fe_3O_4.

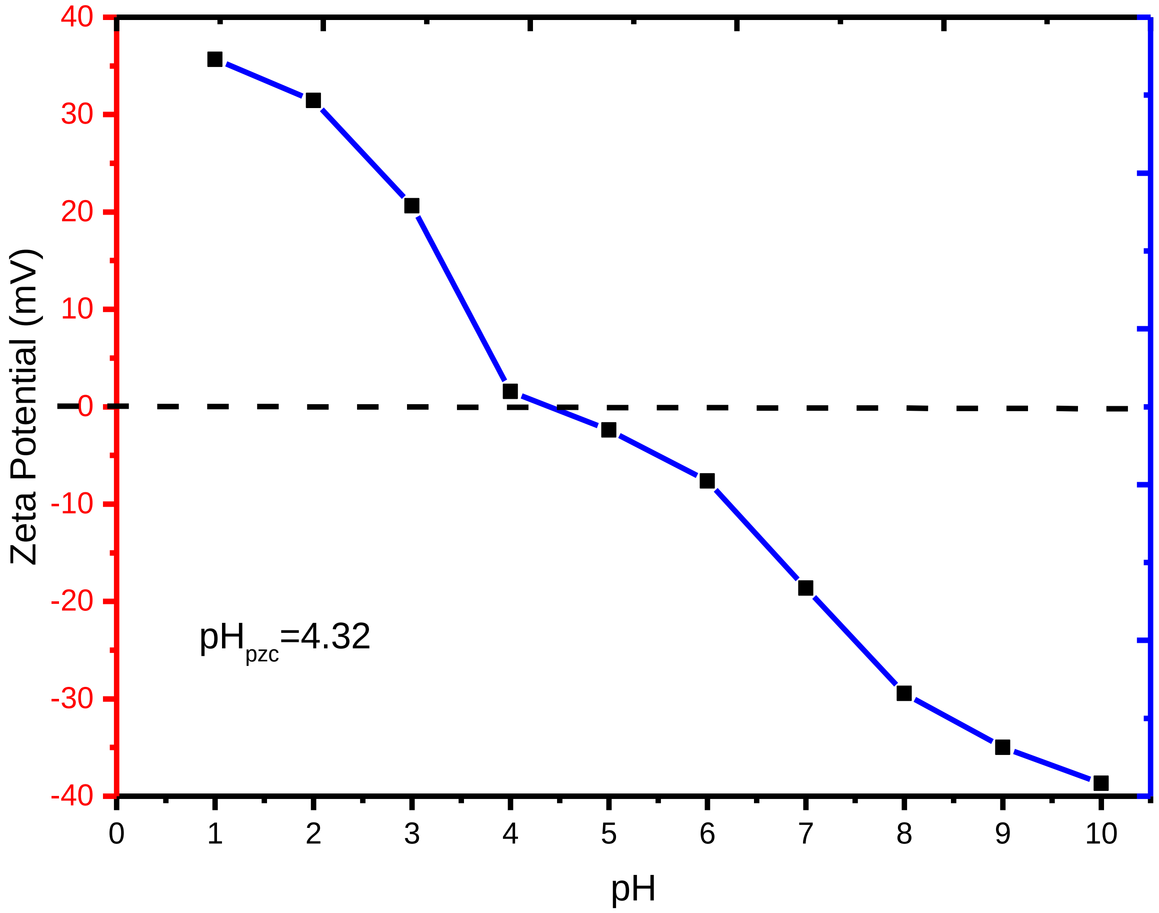

Figure S3. Zeta potential curve for PAC@Fe_3O_4 nanocomposite in a pH range of 1–10 using 0.1 M KCl solution and 100 mg L^{-1} solution of Pb(II).
